# The feasibility of short-segment Schanz screw implanted in an oblique downward direction for the treatment of lumbar 1 burst fracture: a finite element analysis

**DOI:** 10.1186/s13018-020-02024-7

**Published:** 2020-11-17

**Authors:** Jifeng Liu, Sheng Yang, Fei Zhou, Jianmin Lu, Chunyang Xia, Huanhuan Wang, Chao Chen

**Affiliations:** Department of Orthopaedics, Affiliated Zhongshan Hospital of Dalian University, 6 Jiefang Street, Zhongshan District, Dalian, 116001 Liaoning China

**Keywords:** Lumbar burst fracture, Schanz screw, Oblique downward direction, Biomechanics

## Abstract

**Background:**

To evaluate the biomechanical properties of short-segment Schanz screw implanted in an oblique downward direction for the treatment of lumbar 1 burst fracture using a finite element analysis.

**Methods:**

The Universal Spine System (USS) fixation model for adjacent upper and lower vertebrae (T12 and L2) of lumbar 1 vertebra burst fracture was established. During flexion/extension, lateral bending, and rotation, the screw stress and the displacement of bone defect area of the injured vertebrae were evaluated when the downward inserted angle between the long axis of the screws and superior endplate of the adjacent vertebrae was set to 0° (group A), 5° (group B), 10° (group C), and 15°(group D). There were 6 models in each group.

**Results:**

There were no significant differences in the maximum screw stress among all the groups during flexion/extension, lateral bending, and rotation (*P* > 0.05). There were no significant differences in the maximum displacement of the bone defect area of the injured vertebrae among all the groups during flexion/extension, lateral bending, and rotation (*P* > 0.05).

**Conclusion:**

Short-segment Schanz screw implanted in an oblique downward direction with different angles (0°/parellel, 5°, 10°, and 15°) did not change the maximum stress of the screws, and there was a lower risk of screw breakage in all groups during flexion/extension, lateral bending, and rotation. In addition, the displacement of the injured vertebra defect area had no significant changes with the change of angles.

## Background

Burst fractures of the thoracolumbar spine often require surgical treatment; however, there is no uniform standard whether the stenotic spinal canal needs decompression or whether the intraspinal retropulsed bone fragments need to be removed for the fracture combined with nerve injury [1–3]. Miyashita et al. [4] found no significant correlation between nerve recovery and percentage of spinal canal stenosis and provided evidences questioning the need to remove the retropulsed bone fragments in thoracolumbar fractures combined with nerve injury. Therefore, a new method that can effectively treat thoracolumbar burst fractures without decompression of the spinal canal remains to be found in the future.

Short-segment Schanz screw fixation implanted in an oblique downward direction, which was firstly proposed by us in our previous study [1], is a safe and effective method for the treatment of lumbar burst fracture combined with incomplete nerve injury without complications such as screw breakage, screw loosening, and re-collapse of the injured vertebra. Short-segment Schanz screw fixation implanted in an oblique downward direction can achieve an upward and forward reduction of the anterior column, middle column, and the posterior column, thus resulted in a better reduction of the downward and backward retrodisplaced vertebra. Therefore, with this method, the available reduction of burst fractured vertebra (LSC ≥ 7) and spinal canal decompression can be achieved without laminectomy even if when the spinal stenosis was severe, and therefore, it is better than the screw insertion parallel to the endplates, which hardly reduced the intraspinal bone fragments without laminectomy [5, 6].

Although our new method has been clinically successful, the biomechanical properties of a short-segment Schanz screw implanted in an oblique downward direction have not been reported. In this study, the Universal Spine System (USS) instrumentation reduction model for adjacent vertebrae (T12 and L2) of lumbar 1 vertebra burst fracture (LSC ≥ 7) based on load-sharing classification (LSC) [7] was established for the first time using the finite element method. The model was used to evaluate the screw stress and the displacement of the injured vertebrae when the downward inserted angle between the long axis of the screws and superior endplate of the adjacent (T12/L2) vertebrae was set to 0° (group A), 5° (group B), 10° (group C), and 15° (group D), during flexion/extension, lateral bending, and rotation. We hope to verify the feasibility and related biomechanical basis of our method of short-segment Schanz screw instrumentation implanted in an oblique downward direction using the finite element method.

## Methods

The study was approved by the Ethics Committee of Zhongshan Hospital affiliated to Dalian University, and written informed consent was obtained from all volunteers. A total of 6 (5 males and 1 female) healthy young people participated in the experiment. Patients or the public were not involved in the design, conduct, reporting, or dissemination plans of our research. The average age was 27.50 ± 2.51 years old, the height was 175.50 ± 5.92 cm, and the body weight was 76.83 ± 7.70 kg.

### The finite element model

#### Normal T12-L2 model

The DICOM format data of the relevant thoracolumbar images of 6 healthy volunteers were obtained after continuous scanning with 0.75-mm layer thickness using Philips 64-slice spiral computed tomography (CT), followed by Mimics 17.0 (Materialize Inc., Leuven, Belgium), Geomagic studio 2013 (3D Systems, software such as Raindrop Geomagic Inc. USA), and Hyperwork 14.0 (Altair Engineering, Inc., Executive Park, CA, USA) to create the finite element models. Firstly, the CT data in DICOM format were exported to Mimics to obtain a multi-layer continuous image of the coronal, sagittal, and horizontal positions, and the appropriate gray value was set to 275 to highlight the bone structure. The three vertebrae of the T12-L2 segment were subjected to Thresholding, Region Growing, Edit Masks, and Calculate 3D to reconstruct the preliminary three-dimensional geometric model. Subsequently, the model was then imported into the Geomagic studio software in STL format. Grid doctor was used to smooth the surface of the model, repair the holes, and remove the spikes. The model was then fitted to an accurate NURBS surface using the probabilistic curvature method at the exact surface stage. Finally, the NURBS surface was imported into the Hypermesh software in Iges format for meshing, and the corresponding structures were established including the vertebral body, intervertebral disc, and paraspinal ligament. The vertebral body is composed of the cortical bone, cancellous bone, and endplate, and the thickness of the cortical bone and endplate is set to 1 mm. The intervertebral disc consisted of the nucleus pulposus (NP) and annulus fibrosus. The volume ratio of the annulus fibrosus to NP was set to 7:3. The vertebral body is bound to the adjacent intervertebral disc. The thickness of the cortical bone and endplate was set to 1 mm, the thickness of the articular cartilage is 0.3 mm, and the volume of the annulus fibrosus is 50 to 60%. The model was endowed with materials and properties according to previous studies [8, 9], and T3D2 units were used instead of ligaments. The complete normal T12-L2 segment model includes 450,868 ± 55,070 elements and 119,200 ± 13,876 nodes.

#### Internal fixation model

The USS Fracture System model fixation was simulated using Siemens NX 10.0 (Siemens PLM Software, Germany). The fracture clamp was simplified, and the thread was ignored. USS fracture fixation was simulated using Geomagic to complete the model assembly and adjustment of the angle of screw placement. The screw placement was performed using a Roy-Camille method. The screw entrance point should be situated at the crossing of 2 lines on a typical bony crest. The horizontal line should pass through the middle of the transverse process; the vertical line is given by the articular process 1 mm under the facet joint. The internal fixation model is saved as an IGS format file in the same coordinate system as the vertebral model. Mesh was divided by a tetrahedral unit with a side length of 1.5 mm in HyperMesh.

**Fig. 1:**
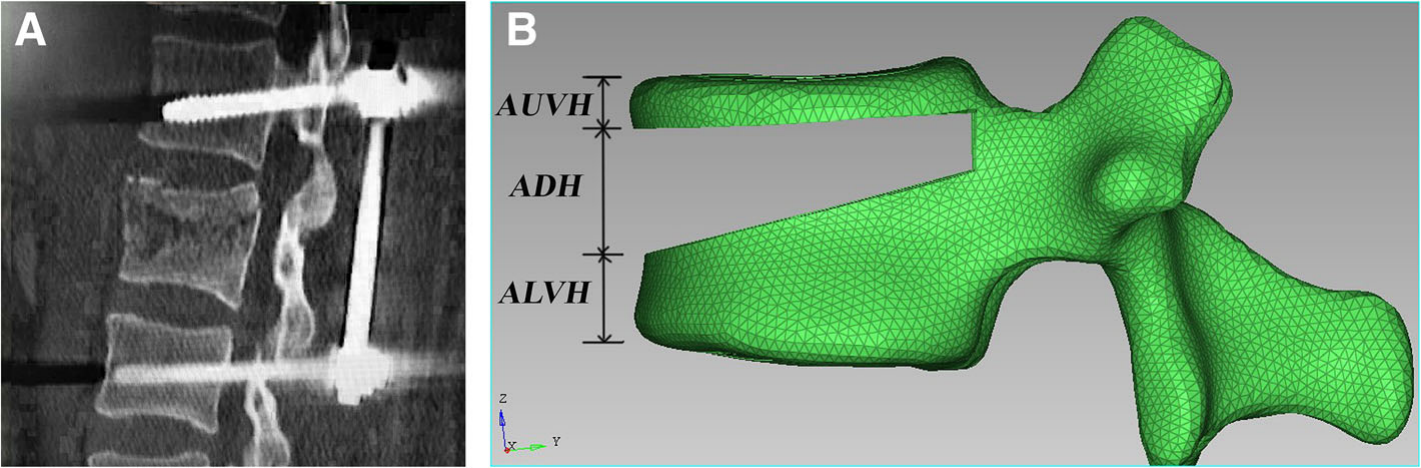
**a** After the reduction of thoracolumbar burst fractures, the wedge-shaped bone defect area in the sagittal position was narrow in the front and wide in the back. **b** AUVH accounts for 15% of the leading edge of the vertebral body, ADH accounts for 50% of the leading edge of the vertebral body, and ALVH accounts for 35% of the leading edge of the vertebral body. AUVH, anterior upper vertebral body height above the bony defect; ADH, anterior bony defect height; ALVH, anterior lower vertebral body height below the bony defect

After the reduction of thoracolumbar burst fractures, the wedge-shaped bone defect area, which was wide in the front and narrow in the back, was found in the sagittal position (Fig. 1a). The methods reported in our previous studies [10] were used to simulate the LSC-based fracture model, with a vertebral anterior column compression of 65% and a kyphotic correction angle of 15° (LSC ≥ 7 points), through wedge resection of part of the normal vertebral body. The ratio of anterior upper vertebral body height above the bony defect (AUVH) = AUVH/AVH(anterior vertebral body height) x 100%; the ratio of anterior bony defect height (ADH) = ADH/AVH × 100%; and the ratio of anterior lower vertebral body height below the bony defect (ALVH) = ALVH/AVH × 100%. The reduction height of the injured vertebra was set to 100%. After reduction, the ratio of AUVH, the ratio of ADH, and the ratio of ALVH were 15%, 50%, and 35%, respectively (Fig. 1b).

**Fig. 2:**
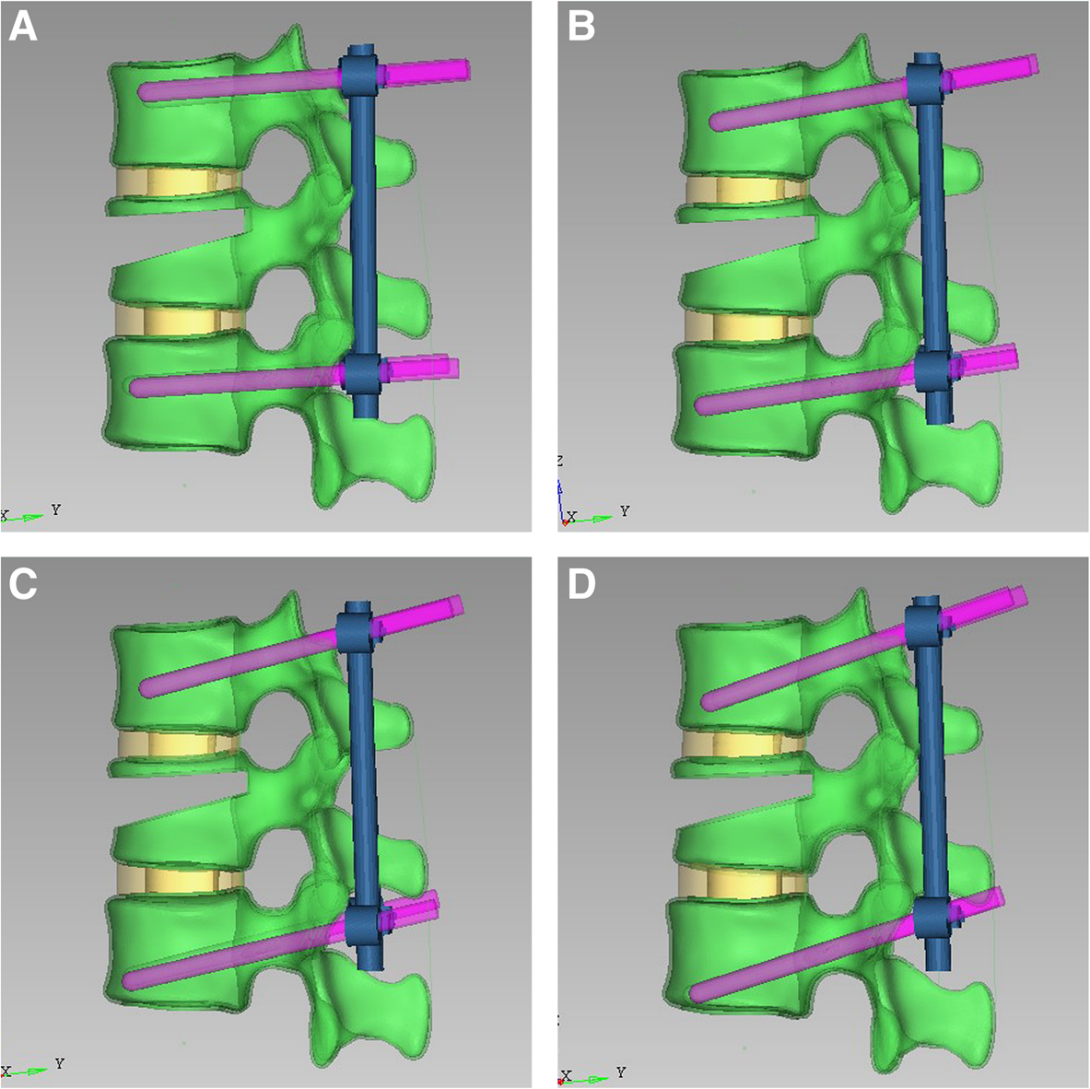
Schanz pedicle screw fixation models when the downward inserted angle between the long axis of the screws and superior endplate of the adjacent vertebrae was set to **a** 0°, **b** 5°, **c** 10°, and **d** 15°

For the four models, the downward inserted angle between the long axis of the screws and superior endplate of the adjacent vertebrae was set to 0° (group A), 5° (group B), 10° (group C), and 15° (group D) (Fig. 2). The screws were all inserted below the anterior cortical bone, and the screw insertion depth accounts for more than 97% of the screw-path length (SPL). The diameters of Schanz pedicle screws and connecting rods are 6.2 mm and 6 mm, respectively [10]. For the 15° model, the inserting position of the screw of the upper and lower screws moved upward by 3.14 ± 1.92 mm and 3.32 ± 0.96 mm, respectively, than that of the other groups to avoid contacting the lower endplate. Assembly was performed using Geomagic, and the junction of the screw and the bone is a continuous mesh and sharing node in Hyperwork.

### Finite element analysis

We simulated 4 groups according to the downward inserted angle between the long axis of the screws and superior endplate of the adjacent vertebrae, 0° of group A (parallel to the endplate) and other three groups all in an oblique downward: respectively 5°, 10°, and 15°. There are 6 models in each group.

The concentrated downward pressure along the *Z*-axis direction (vertical load) applied to the upper surface of the T12 vertebral body was used to simulate the gravity of the human body when standing upright. The torque applied is a rotating torque that makes the vertebral body rotate in an axial direction, which was used to simulate the role of the paravertebral muscles in driving the spine to perform various activities including flexion/extension, lateral bending, and rotation [11–13]:

1. Vertical loads of 350 N.
2. Torque of 7.5 Nm during flexion/extension, lateral bending, and rotation, and a vertical load of 350 N.

### Statistical analysis

Statistical analysis was performed using the SPSS 20.0 software (IBM, USA). Values are presented as mean ± standard deviation (SD). Data between the different groups during flexion/extension, lateral bending, and rotation were compared by one-way ANOVA. A *P* value of less than 0.05 was considered statistically significant.

## Results

### Validation of the model

In this study, the range of motion (ROM) of T12/L1 and L1/L2 vertebra of the normal T12-L2 model during flexion/extension, lateral bending, and rotation was similar to those of Yamamoto et al. [14] and Panjabi et al. [15]. Therefore, the T12-L2 model in this study was valid for further analyses.

### Pedicle screw stress

**Fig. 3:**
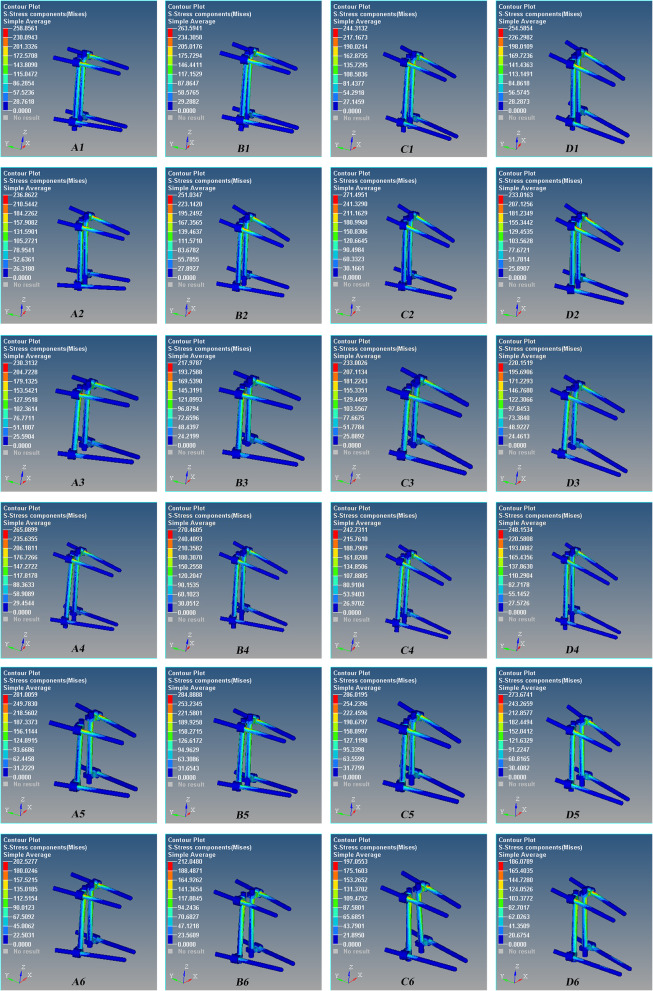
The stress nephogram of the Schanz pedicle screw for the L1 severe fractures after T12 and L2 pedicle screw fixation during anterior flexion. Red is the maximum stress. The maximum stress occurred at the interface of the proximal pedicle and cortical bone, and the stress of the upper screw is greater than that of the lower screw. **A1** 0°, **B1** 5°, **C1** 10°, and **D1** 15°. One model in each group was shown

**Fig. 4:**
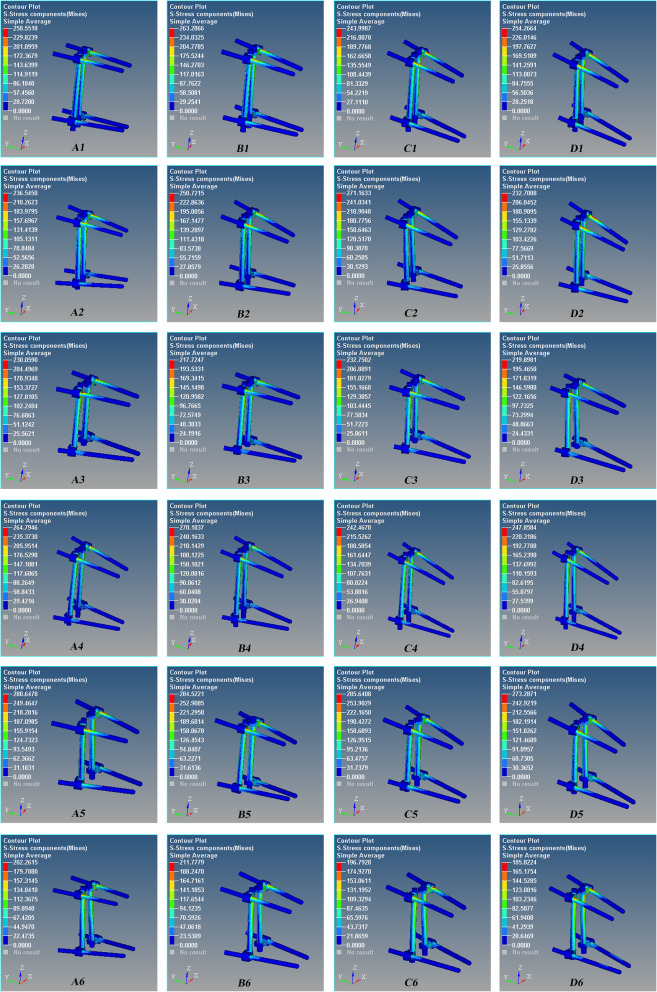
The stress nephogram of the Schanz pedicle screw for the L1 severe fractures after T12 and L2 pedicle screw fixation during posterior extension. Red is the maximum stress. The maximum stress occurred at the interface of the proximal pedicle and cortical bone, and the stress of the upper screw is greater than that of the lower screw. **A1** 0°, **B1** 5°, **C1** 10°, and **D1** 15°. One model in each group was shown

**Fig. 5:**
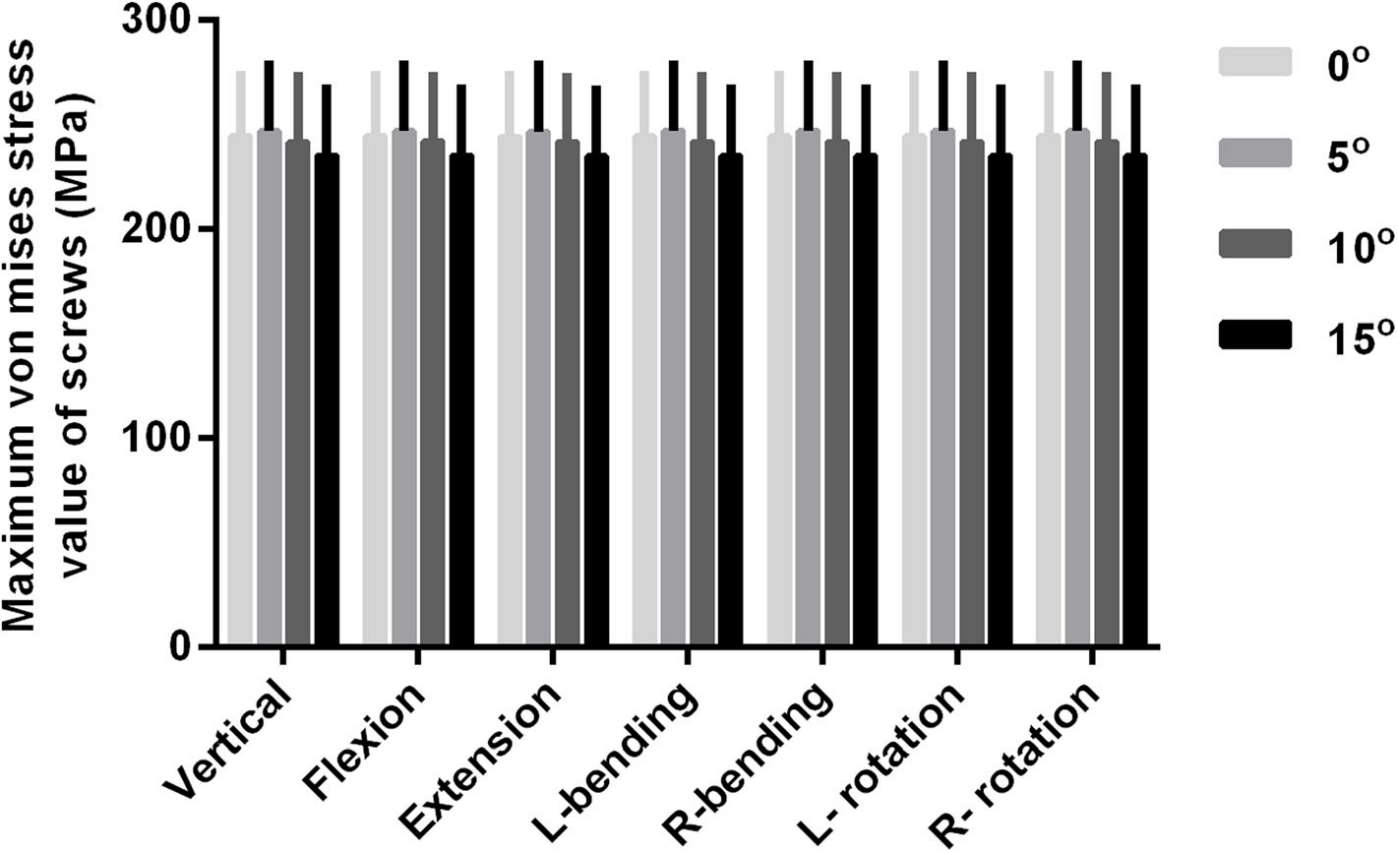
Comparison of the maximum stress of the screws when the downward inserted angle between the long axis of the screws and superior endplate of the adjacent vertebrae was set to 0° (group A), 5° (group B), 10° (group C), and 15° (group D) during flexion/extension, lateral bending, and rotation. The maximum stress of the screws has no statistical difference among the four groups (*P* > 0.05)

The maximum stress occurred at the interface of the proximal pedicle and cortical bone, and the stress of the upper screw is greater than that of the lower screw (Figs. 3 and 4). The maximum stress of the screws had no statistical difference among the four groups (*P* > 0.05, Fig. 5).

### Postoperative axial displacement/micro-motion of the lumbar 1

**Fig. 6:**
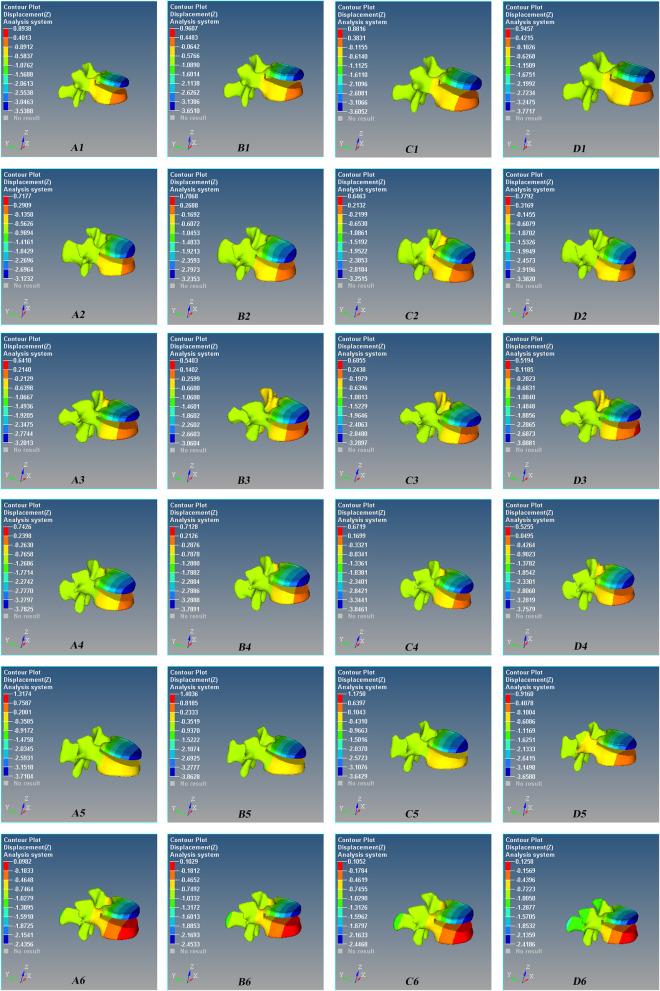
Axial (*Z*-axis) displacement nephogram of the micro-motion of the vertebral defect area of Schanz pedicle screw for L1 severe fracture during anterior flexion. Red is the maximum displacement upward, and blue is the maximum displacement downward. **A1** 0°, **B1** 5°, **C1** 10°, and **D1** 15°. One model in each group was shown

**Fig. 7:**
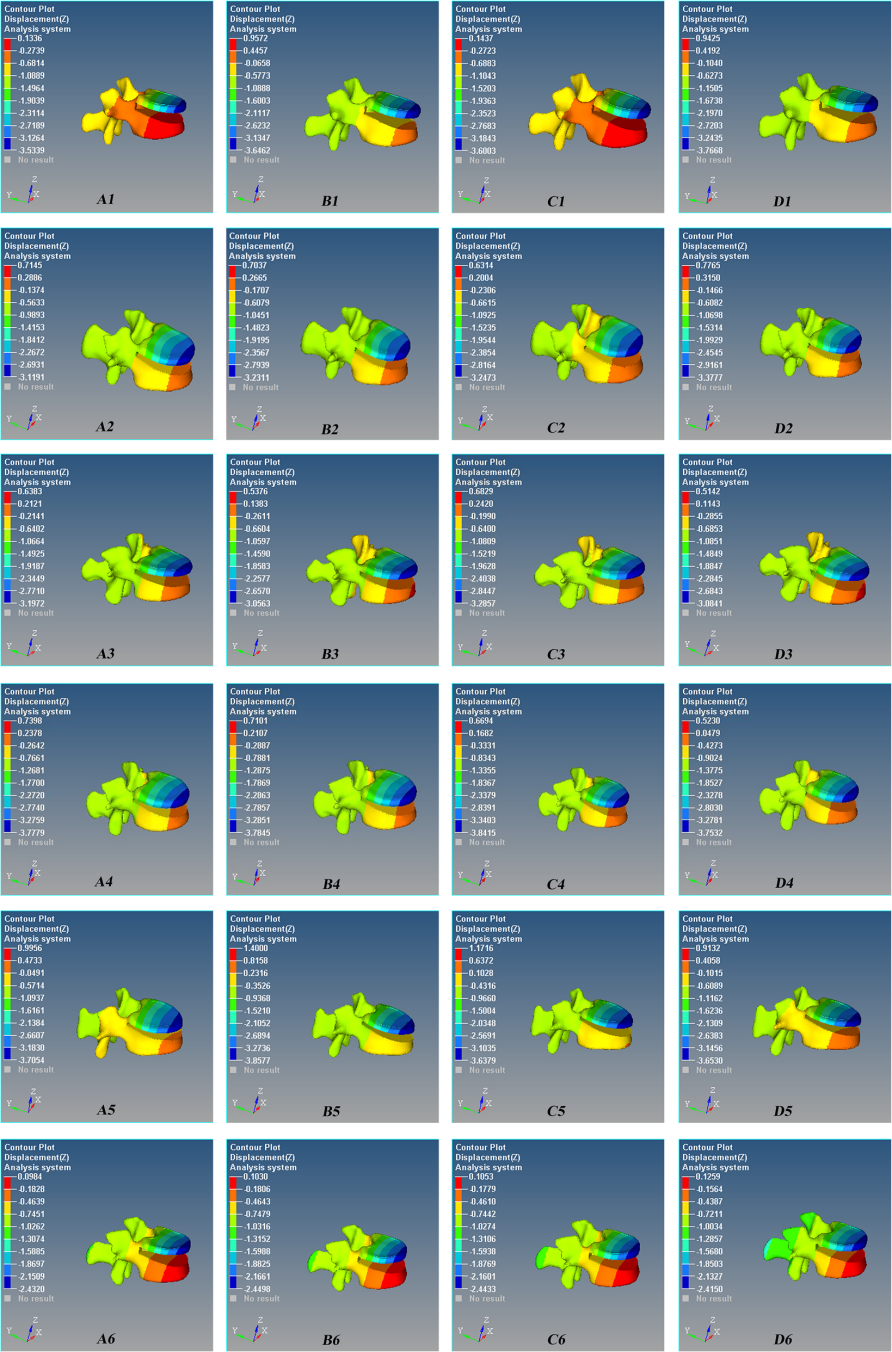
Axial (*Z*-axis) displacement nephogram of the micro-motion of vertebral defect area of Schanz pedicle screw for L1 severe fracture during posterior extension. Red is the maximum displacement upward, and blue is the maximum displacement downward. **A1** 0°, **B1** 5°, **C1** 10°, and **D1** 15°. One model in each group was shown

**Fig. 8:**
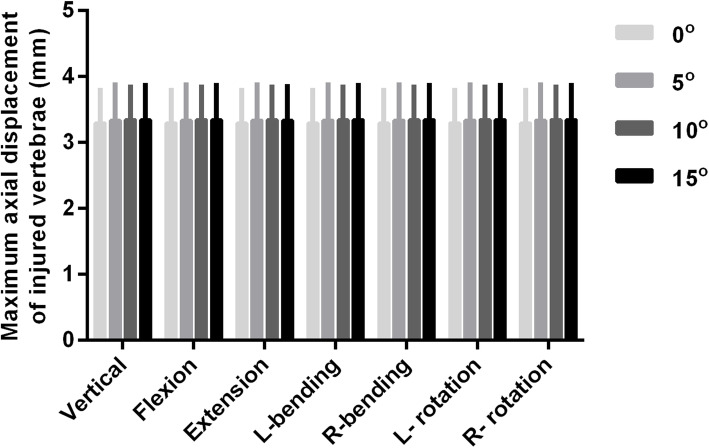
Comparison of the maximum axial (*Z*-axis) displacement of the vertebral defect area when the downward inserted angle between the long axis of the screws and superior endplate of the adjacent vertebrae was set to 0° (group A), 5° (group B), 10° (group C), and 15° (group D) during flexion/extension, lateral bending, and rotation. The axial displacement of the injured vertebrae increased with the increase of the angle during flexion/extension, lateral bending, and rotation, but there was no statistical difference among the four groups (*P* > 0.05)

The displacement of the injured vertebra was more in the leading edge and less in the trailing edge. There was a “cohesive” displacement/micro-motion between the upper vertebral body and the lower vertebral body of lumbar 1 due to the downward displacement/micro-motion of the upper vertebral body and the upward displacement/micro-motion of the lower vertebral body (Figs. 6 and 7). The axial displacement of the injured vertebrae increased with the increase of the angle during flexion/extension, lateral bending, and rotation, but there was no statistical difference among the four groups (*P* > 0.05, Fig. 8).

## Discussion

Previous finite element simulation of the fracture models is not widely accepted and is different from clinical practice due to the removal of the part of the cortical or cancellous bone, which is not suitable for comparative analysis of various finite element studies [11, 16, 17]. LSC is a widely accepted vertebral body load scoring standard for the treatment of thoracolumbar fractures. The LSC system is a straightforward, quantitative method of describing the following 3 aspects: (1) extent of bony comminution, (2) amount of fracture displacement, and (3) extent of kyphosis in a vertebra fracture, which can well meet the requirements of finite element for quantitative simulation of type A fractures. Each aspect was recorded as 1 point (mild), 2 points (moderate), and 3 points (severe) according to the fracture severity. The most severe fracture was calculated as a maximum of 9 points. In 2018, De Iure et al. [18] retrospectively analyzed 121 cases of unstable thoracolumbar fractures and concluded that the LSC-based injured vertebral assessment can predict the risk of posterior fixation failure. Based on LSC, our previous finite element analysis compared short-segment instrumentation with conventional pedicle screws and the Schanz pedicle screw in lumbar 1 fractures, indicating that Schanz pedicle screws were recommended for unstable fractures because the screws have a lower risk of screw breakage compared with conventional pedicle screws [10].

In order to better evaluate the maximum stress of screws and the displacement of the injured vertebra defect area when short-segment Schanz screws were implanted in an oblique downward direction with different angles, the following settings were made using the LSC-based injured vertebra model. Healthy young people have less degeneration and proliferation of thoracolumbar spine and intervertebral disc, and the spine is easier to simulate and the model is closer to reality compared with the degenerated spine (vertebral osteophytes, elastic changes of ligaments, degeneration of intervertebral discs, etc.). Therefore, our study is based on six healthy young people. The height restoration compression of the vertebral body was set to be 100%. The anterior edge compression of the vertebra was 65% (3 points), the kyphotic correction angle was 15° (2 points), and the fracture extent and the displacement of broken bone were 1–3 points; therefore, the total LSC was 7–9 points. Similar to our previous study [10, 19], the bone defect area was a triangular-like bony defect which was wide in the front and narrow in the back. In addition, the upper vertebral body of the bony defect retains 15% of the bone for analysis.

Using the above model, four groups were set in our study according to the downward inserted angle between the long axis of the screws and superior endplate of the adjacent vertebrae: 0° of group A, 5° of group B, 10° of group C, and 15° of group D. The results of the comparative analysis showed that there was no significant difference in the maximum stress among the groups during flexion/extension, lateral bending, and rotation with the increase of the angle (*P* = 0.92). The maximum stress of each group was lower than 250 MPa, which is far below the fatigue threshold of 550 MPa [20], indicating a lower risk of screw breakage in all groups during all these situations. And this is consistent with our previous clinical results which showed that Schanz screw downward fixation is safe and effective in the treatment of lumbar burst fracture [1].

The effect of short-segment pedicle screw fixation for thoracolumbar spine fractures is load-bearing and trans-vertebral stress conduction. In our previous study [1], we proposed that the conventional pedicle screw has a similar load-bearing capacity to the Schanz screw; however, the latter has a better conduction capacity because the structure of the Schanz screw rod (similar to “][”-shaped conduction) is more similar to the lumbar posterior column (butterfly-shaped conduction) than conventional pedicle screw (similar to “| |”-shaped conduction). In addition, we first proposed that the difference in conduction ability is the key to the screw breakage and that is why the Schanz screw fixation can effectively treat severe burst fractures of the thoracolumbar segment than conventional screw fixation. Therefore, the nonsignificant differences in the maximum stress among the groups in the present study suggest that the stress conduction does not change significantly with the change of the oblique angle. Similar to other studies [13, 16, 21], the maximum stress of screws in each group in this study was located in the upper screws during anterior flexion, which was mainly because the stress during anterior flexion was conducted from the upper pedicle screw to the lower pedicle screw across the injured vertebra and adjacent intervertebral disc.

Despite no screw breakage occurred in Schanz pedicle screw fixation for the treatment of severe fracture [1, 2], it still has the disadvantages of postoperative re-collapse of the injured vertebra [22]. Jang et al. retrospectively analyzed 208 cases of thoracolumbar burst fracture using conventional open pedicle screw fixation and found that age at operation (> 43 years old) and preoperative body height loss (> 54%) were independent predictors of re-collapse [23]. In our previous studies, we found that the compression displacement/micro-motion of the bony defect of injured vertebrae during flexion may contribute to postoperative re-collapse of the injured vertebrae, and the “cohesive” displacement/micro-motion of the bony defect of unstable fractures was larger than that of moderate fractures under the same screws [1, 19]. However, to the best of our knowledge, there were few finite element studies simulating and evaluating the bone defect of the injured vertebra after reduction [11, 17, 24].

In this present study, there was also a “cohesive” displacement/micro-motion of the bone defect area; however, the change of the oblique angle did not change the displacement/micro-motion of the bone defect area. By the combined use of Schanz pedicle screw and thoraco-lumbo-sacral orthosis (TLSO) brace in our previous study [1] and the study of Aono et al. [2], we not only ensured the clinical efficacy but also prevented the re-collapse of the injured vertebra. We speculated that TLSO brace could limit the thoracolumbar sacral movement to reduce the injured vertebra displacement and thus avoid the re-collapse of the injured vertebrae [1]; however, further biomechanical and clinical studies are needed to confirm this statement.

Short-segment Schanz screw implanted in an oblique downward direction with different angles did not change the maximum stress of screws and the displacement of the injured vertebra defect area, the following deficiencies need to be further verified: (1) There were 6 samples in each group in our study, and more samples are needed to confirm this finding, and studies on the stress of the pedicle screw based on middle-aged and older people remain to be further studied. (2) Whether the biomechanical results of related cadaver models are consistent. (3) The anti-collapse effect of the TLSO brace needs further exploration.

## Conclusion

In conclusion, short-segment Schanz screw implanted in an oblique downward direction with different angles did not significantly change the maximum stress of screws and the displacement of the injured vertebra defect area. This is a safe method to treat severe L1 burst fracture.

## Abbreviations

USS: Universal Spine System
CT: Computed tomography
NP: Nucleus pulposus
AUVH: Anterior upper vertebral body height above the bony defect
AVH: Anterior vertebral body height
ADH: Anterior bony defect height
ALVH: Anterior lower vertebral body height below the bony defect
ROM: Range of motion
TLSO: Thoraco-lumbo-sacral orthosis
