# Correction to: The feasibility of short-segment Schanz screw implanted in an oblique downward direction for the treatment of lumbar 1 burst fracture: a finite element analysis

**DOI:** 10.1186/s13018-020-02134-2

**Published:** 2020-12-24

**Authors:** Jifeng Liu, Sheng Yang, Fei Zhou, Jianmin Lu, Chunyang Xia, Huanhuan Wang, Chao Chen

**Affiliations:** Department of Orthopaedics, Affiliated Zhongshan Hospital of Dalian University, 6 Jiefang Street, Zhongshan District, Dalian, 116001 Liaoning China

**Correction to: J Orthop Surg Res (2020) 15:537**

**https://doi.org/10.1186/s13018-020-02024-7**

Following publication of the original article [1], due to a typesetting error, the figure legends for Figs. 1, 3, 4, 6 and 7 were wrongly published. The correct figure legends are given below.

**Fig. 1:**
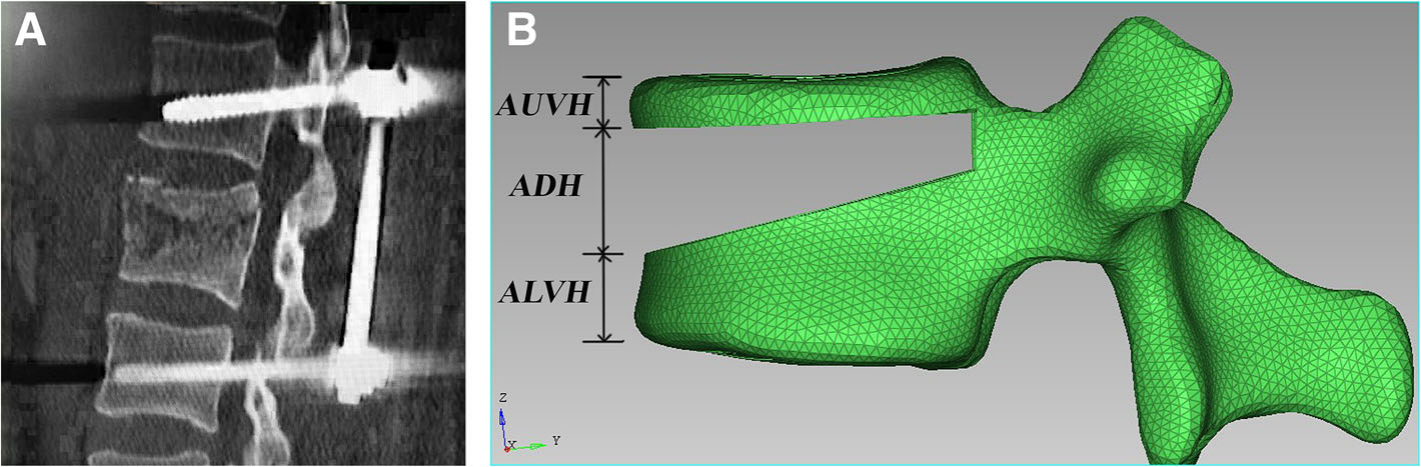
**a** After the reduction of thoracolumbar burst fractures, the wedge-shaped bone defect area in the sagittal position was wide in the front and narrow in the back. **b** AUVH accounts for 15% of the leading edge of the vertebral body, ADH accounts for 50% of the leading edge of the vertebral body, and ALVH accounts for 35% of the leading edge of the vertebral body. AUVH, anterior upper vertebral body height above the bony defect; ADH, anterior bony defect height; ALVH, anterior lower vertebral body height below the bony defect

**Fig. 3:**
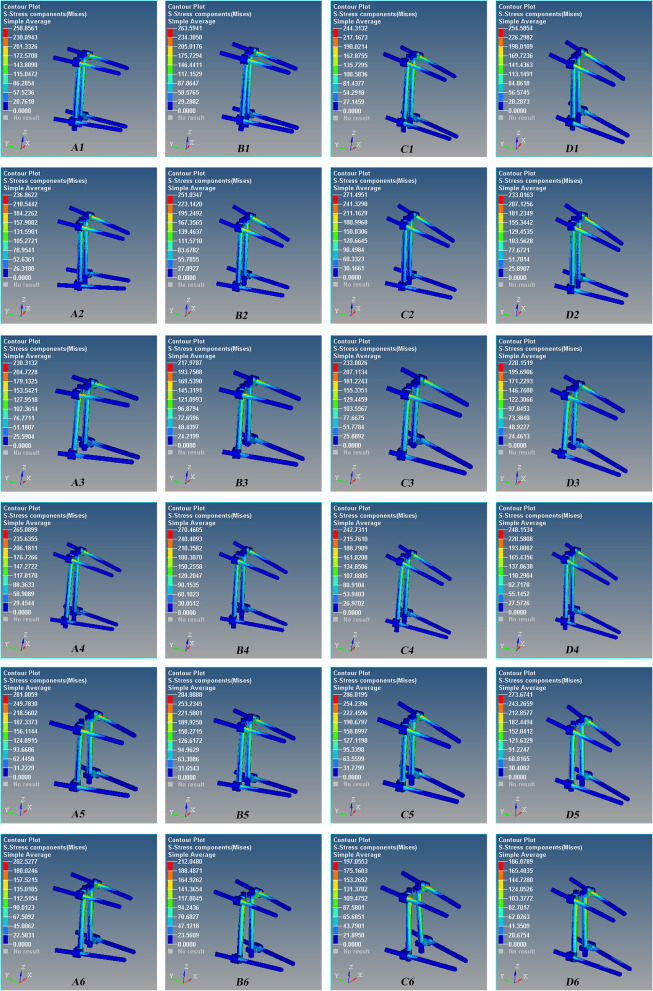
The stress nephogram of the Schanz pedicle screw for the L1 severe fractures after T12 and L2 pedicle screw fixation during anterior flexion. Red is the maximum stress. The maximum stress occurred at the interface of the proximal pedicle and cortical bone, and the stress of the upper screw is greater than that of the lower screw. A1–A6 0°, B1–B6 5°, C1–C6 10°, and D1–D6 15°. There are 6 models in each group

**Fig. 4:**
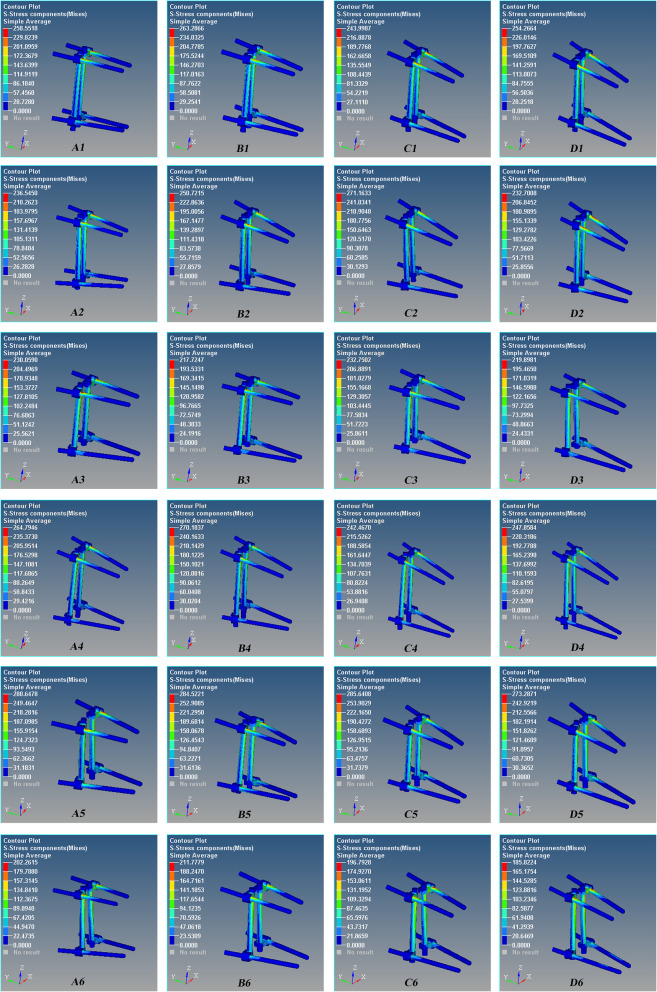
The stress nephogram of the Schanz pedicle screw for the L1 severe fractures after T12 and L2 pedicle screw fixation during posterior extension. Red is the maximum stress. The maximum stress occurred at the interface of the proximal pedicle and cortical bone, and the stress of the upper screw is greater than that of the lower screw. A1–A6 0°, B1–B6 5°, C1–C6 10°, and D1–D6 15°. There are 6 models in each group

**Fig. 6:**
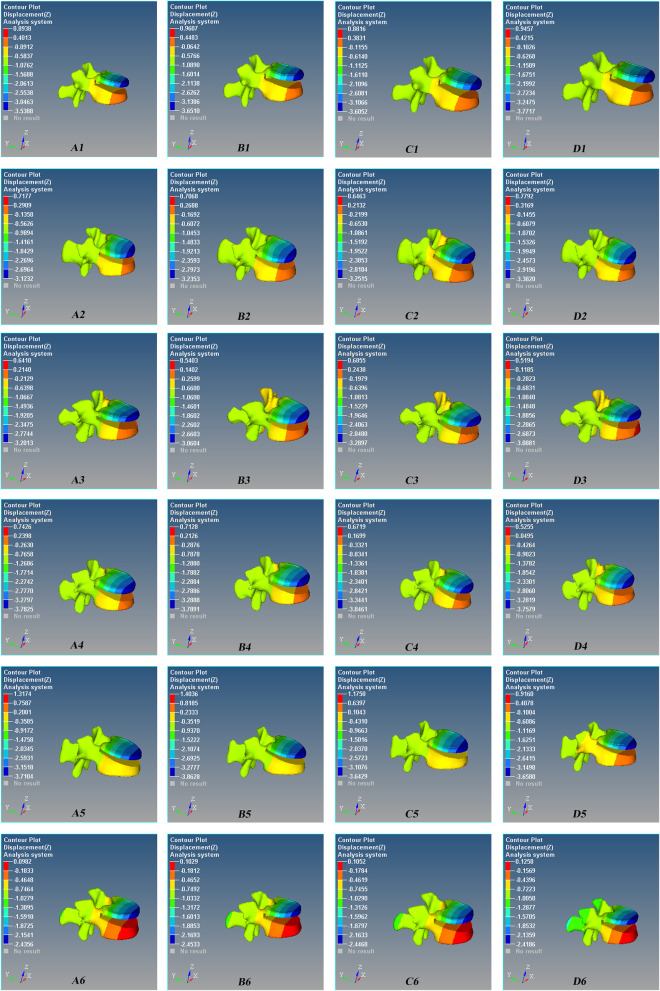
Axial (Z-axis) displacement nephogram of the micro-motion of the vertebral defect area of Schanz pedicle screw for L1 severe fracture during anterior flexion. Red is the maximum displacement upward, and blue is the maximum displacement downward. A1–A6 0°, B1–B6 5°, C1–C6 10°, and D1–D6 15°. There are 6 models in each group

**Fig. 7:**
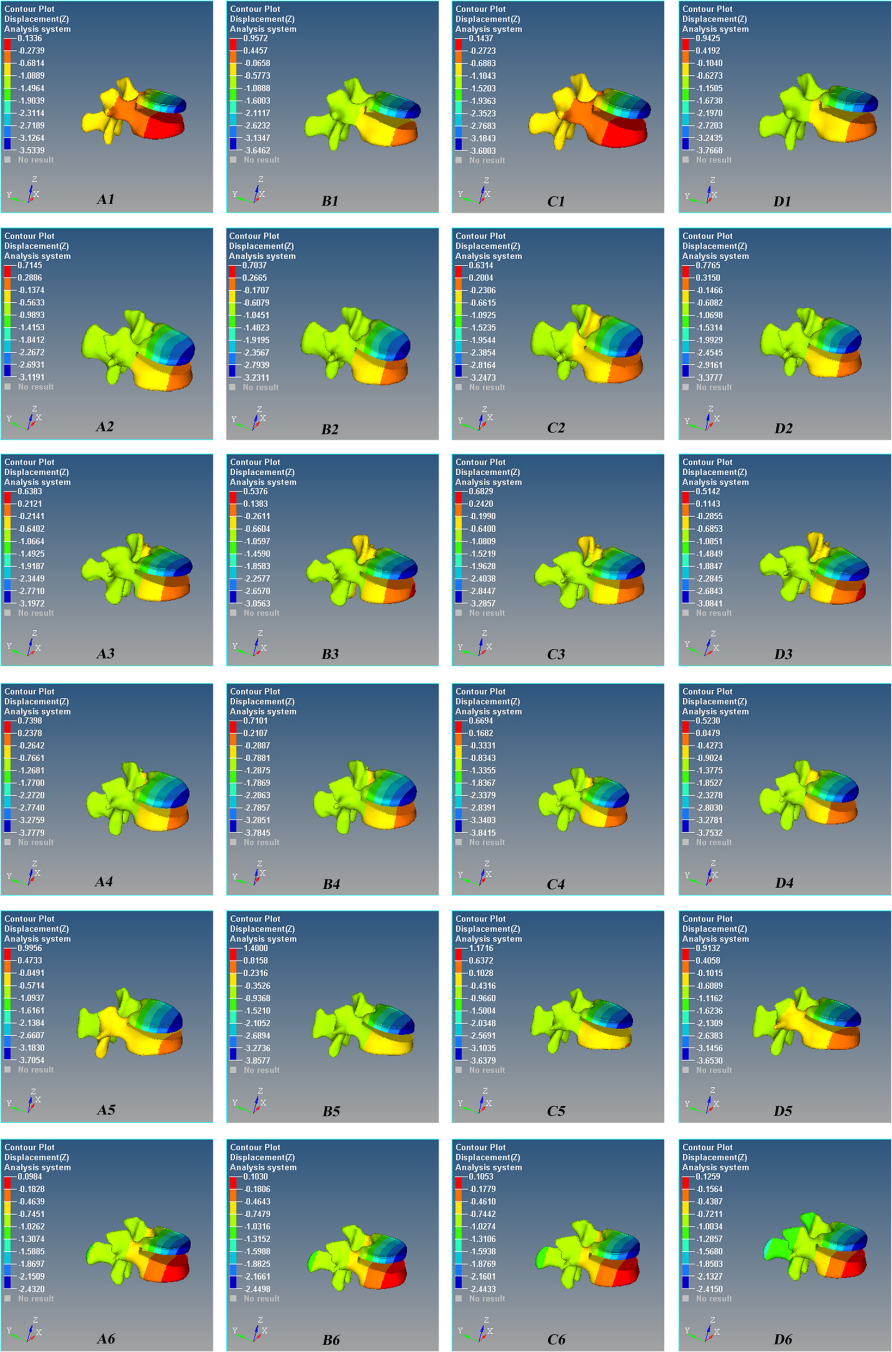
Axial (Z-axis) displacement nephogram of the micro-motion of vertebral defect area of Schanz pedicle screw for L1 severe fracture during posterior extension. Red is the maximum displacement upward, and blue is the maximum displacement downward. A1–A6 0°, B1–B6 5°, C1–C6 10°, and D1–D6 15°. There are 6 models in each group

The authors would also like to correct the citation form page 10.

Incorrect: [1, 19]

Correct: [10]

The original article has been corrected.
